# Metabolic dysfunction-associated steatotic liver disease and immune checkpoint inhibitor response in gastrointestinal liver metastases: current evidence and future directions

**DOI:** 10.3389/fnut.2026.1878927

**Published:** 2026-07-29

**Authors:** Shilin Guo, Bohan Liu, Heng Li, Yichen Yu, Jianwei Xu

**Affiliations:** Department of Pancreatic Surgery, General Surgery, Qilu Hospital of Shandong University, Jinan, China

**Keywords:** gastrointestinal cancer, immune checkpoint inhibitors, liver metastasis, MASH, MASLD, tumor microenvironment

## Abstract

Liver metastasis is associated with reduced benefit from immune checkpoint inhibitors (ICIs) in several gastrointestinal cancers, yet the background liver status is rarely considered in immunotherapy biomarker studies. Here, we examine whether metabolic dysfunction-associated steatotic liver disease (MASLD) and metabolic dysfunction-associated steatohepatitis (MASH) may alter the immune environment of liver metastases and affect ICI efficacy. The available evidence is strongest, and most mechanistic, for colorectal liver metastasis and derives largely from preclinical models; it is emerging for pancreatic ductal adenocarcinoma and limited for gastric and biliary tract cancers. MASLD and MASH should not be treated as interchangeable: simple steatosis, active steatohepatitis, fibrosis, and cirrhosis represent distinct liver conditions with different possible effects on hepatic seeding and immune-cell access. Early steatohepatitic inflammation may promote hepatic colonization through lipid availability, hepatocyte stress, and myeloid-cell recruitment, whereas advanced fibrosis can reduce initial colonization while impairing lymphocyte access to established lesions. ICI efficacy requires viable tumor-reactive T cells, relief from myeloid-cell suppression, and direct tumor-T-cell contact; MASLD- and MASH-related liver changes may interfere with each of these requirements through mechanisms beyond checkpoint-ligand expression alone. We distinguish T-cell exhaustion from liver metastasis-associated T-cell deletion, and highlight macrophage-mediated elimination of tumor-specific CD8-positive T cells, myeloid chemokine signaling-driven suppressor-cell recruitment, and stromal remodeling as candidate mechanisms, supported mainly by preclinical evidence, that may reduce ICI response. These mechanisms remain unvalidated as predictors of ICI response in cohorts with MASLD/MASH staging. Future studies should assess liver fat, inflammatory activity, fibrosis stage, tumor genotype, and ICI outcomes in the same patients to test whether MASLD/MASH stage is associated with differential benefit.

## 1 Introduction

Liver metastasis is not only a manifestation of gastrointestinal tumor spread; it also develops within a hepatic immune environment that can affect treatment response (1–3). Immune checkpoint inhibitors (ICIs) have improved outcomes in selected gastrointestinal cancer subgroups, especially in microsatellite instability-high or mismatch repair-deficient metastatic colorectal cancer. However, many patients with liver metastases still have limited or short-lived benefit from ICIs (2, 4–6). These findings suggest that immunotherapy response is influenced not only by tumor-intrinsic factors, but also by the liver tissue in which metastatic tumors grow.

The liver has a unique immune environment. It is continuously exposed to portal venous antigens, microbial products, and gut-derived inflammatory signals, which contributes to baseline immune tolerance (3). Metastatic tumors may take advantage of this tolerant environment to evade antitumor immunity. Experimental and clinical studies have shown that liver metastases can reduce the efficacy of immune checkpoint blockade by attracting tumor-specific CD8-positive T cells to the liver and promoting their macrophage-mediated elimination (2). This may partly explain why liver metastases are associated with poorer ICI outcomes, and why liver status should be measured in immunotherapy trials (5).

Metabolic dysfunction-associated steatotic liver disease (MASLD) and metabolic dysfunction-associated steatohepatitis (MASH) can further modify the liver environment in patients with liver metastases. MASLD refers to steatotic liver disease associated with cardiometabolic risk factors, whereas MASH refers to the inflammatory steatohepatitic form of this disease spectrum (7, 8). These conditions can alter the liver through changes in lipid accumulation, inflammation, and fibrosis (8). Despite this, MASLD and MASH are rarely measured as baseline liver conditions in immunotherapy trials, biomarker studies, or studies of liver metastasis.

Here, we examine whether MASLD- and MASH-related liver changes alter immune features of gastrointestinal liver metastases and contribute to reduced ICI response. The focus is on conditions needed for ICI efficacy, including effector T-cell survival, relief from myeloid-cell suppression, and lymphocyte access to metastatic tumor cells. MASLD and MASH are not presented as established biomarkers for ICI selection, but as background liver conditions that may affect the immune behavior of liver metastases. We separate direct clinical evidence from translational studies, animal data, and possible mechanisms to avoid overstatement. Colorectal cancer and emerging pancreatic ductal adenocarcinoma data are discussed separately from gastric, gastroesophageal, and biliary tract cancers, where MASLD/MASH-stratified evidence remains limited (9).

**Figure 1:**
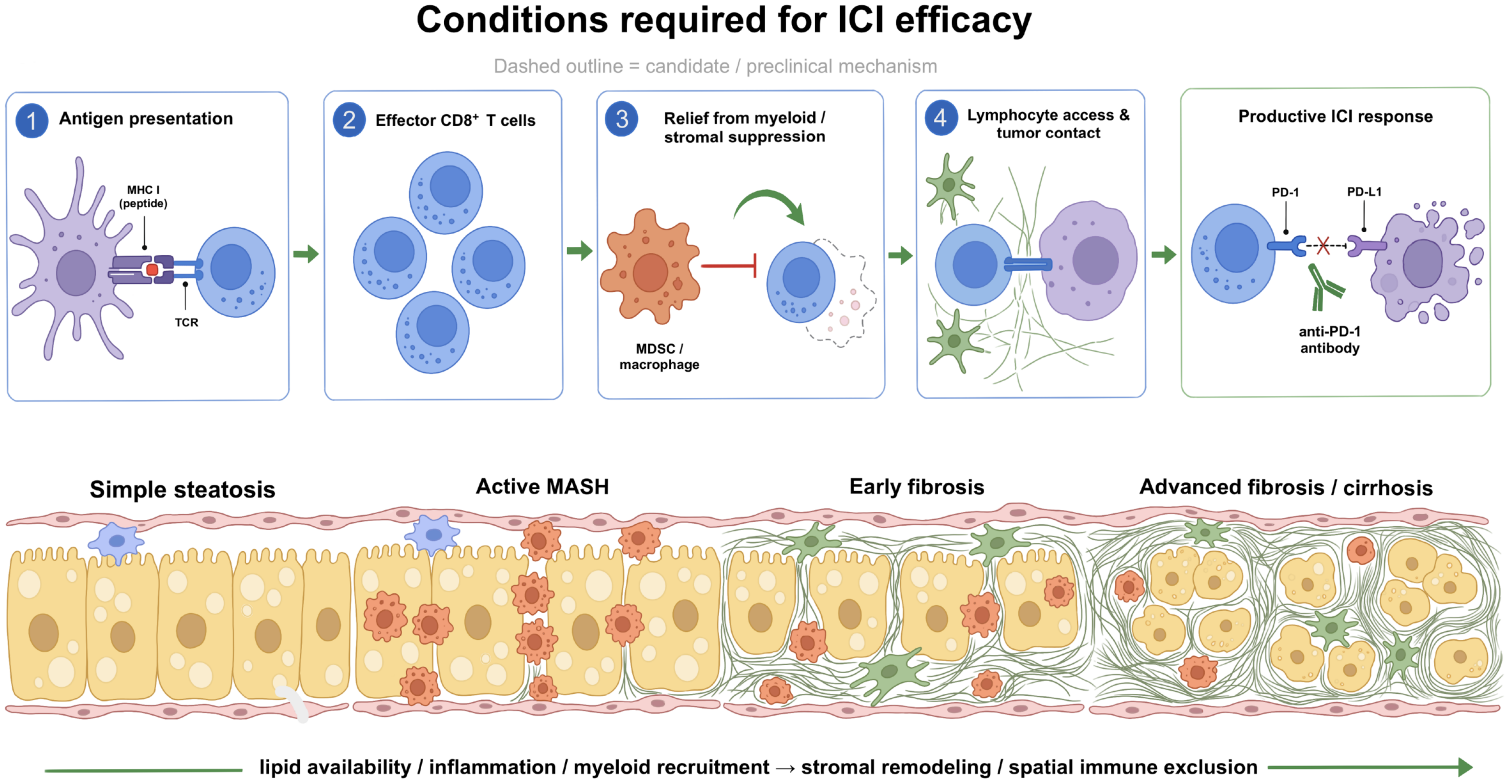
Conditions required for immune checkpoint inhibitor efficacy and the background-liver disease-stage context. Checkpoint blockade does not act on tumor cells directly; it depends on four sequential requirements (steps 1–4), shown left to right: (1) Antigen presentation, in which a peptide presented on MHC class I is recognized by the T-cell receptor of a CD8-positive T cell; (2) The presence of viable effector CD8-positive T cells; (3) Relief from myeloid- and stromal-mediated suppression, illustrated by MDSC/macrophage inhibition of a T cell; and (4) Physical access of lymphocytes to malignant cells, in which a T cell penetrates the stromal mesh to make direct tumor–T-cell contact. When these requirements are met, a productive ICI response occurs, shown as CD8-positive T-cell killing of a tumor cell with an anti-PD-1 antibody blocking the PD-1–PD-L1 interaction. The lower band shows the corresponding background-liver disease-stage axis—Simple steatosis, active MASH, early fibrosis, and advanced fibrosis/cirrhosis—progressing from lipid availability, inflammation, and myeloid recruitment toward stromal remodeling and spatial immune exclusion. Dashed outlines denote candidate or preclinical mechanisms; these derive predominantly from preclinical and murine models and have not been validated in human gastrointestinal liver-metastasis cohorts staged for MASLD/MASH. APC, antigen-presenting cell; CD8, cluster of differentiation 8; DC, dendritic cell; ICI, immune checkpoint inhibitor; MASH, metabolic dysfunction-associated steatohepatitis; MASLD, metabolic dysfunction-associated steatotic liver disease; MDSC, myeloid-derived suppressor cell; MHC, major histocompatibility complex; PD-1, programmed cell death protein 1; PD-L1, programmed death-ligand 1; TCR, T-cell receptor.

Effective checkpoint blockade does not act on tumor cells directly; it depends on a chain of immune requirements. Throughout this review we organize the evidence around four conditions necessary for ICI benefit: (i) adequate tumor-antigen presentation; (ii) the presence and survival of tumor-reactive effector CD8-positive T cells; (iii) relief from dominant myeloid- and stromal-mediated suppression; and (iv) physical access of cytotoxic lymphocytes to malignant cells. MASLD- and MASH-related liver changes are then mapped onto these four requirements rather than treated as a single, undifferentiated ‘fatty-liver’ effect. This framework, and the points at which metabolic liver disease may interrupt each requirement, is summarized in Figure 1.

Search strategy. This is a narrative review. We searched PubMed/MEDLINE and Web of Science for English-language articles published up to 1 May 2026, combining terms for MASLD/MASH/NAFLD/NASH/hepatic steatosis, liver metastasis, gastrointestinal cancer (colorectal, pancreatic, gastric/gastroesophageal, biliary), the hepatic immune microenvironment, and immune checkpoint inhibitors/PD-1/PD-L1. Reference lists of key articles and recent guidelines were screened for additional sources. Given the narrative scope, studies were selected for conceptual relevance and methodological quality rather than by a predefined systematic protocol; preclinical, translational, and clinical sources are graded by evidence level in Table 1.

**Table 1:** Evidence on MASLD/MASH-related liver changes, liver metastases, and ICI response.

Evidence level	Main evidence	Representative evidence	Interpretation
Direct clinical evidence	Liver metastasis is associated with reduced ICI benefit in several clinical studies. ICI outcome data by MASLD/MASH stage are still lacking.	Yu et al. (2); Chen et al. (6); Tian et al. (17)	Supports liver metastasis as a clinically relevant factor in ICI response, but does not establish MASLD/MASH stage as an ICI biomarker.
Translational human evidence	Steatotic liver tissue may affect liver metastases through lipid transfer, hepatocyte-derived vesicles, stromal remodeling, and immune-cell localization.	Li et al. (27); Wang et al. (29); Yang et al. (59); Wu et al. (69)	Supports a possible link between fatty liver and changes in liver metastases, but does not prove that MASLD/MASH stage predicts ICI resistance.
Animal-model evidence	Diet-induced fatty liver or steatohepatitis can affect metastatic growth, myeloid-cell recruitment, macrophage polarization, and anti-PD-1 response in selected models.	Yang et al. (30); Ohashi et al. (33); Yang et al. (39)	Supports possible reasons for reduced ICI response, but the evidence remains preclinical.
Mechanistic evidence	NET-related changes, stromal remodeling, and immune-cell localization are linked to impaired immune-cell access and reduced T-cell activity in MASLD/MASH-associated liver metastases.	Yang et al. (38); HGP consensus (51, 52); immune-localization studies (53–57)	Relevant to future studies, but not evidence that these pathways predict ICI response in patients staged for MASLD/MASH.
Potential treatment approaches	Metabolic, myeloid-targeted, NET-related, and matrix-targeted approaches require patient selection by liver stage, tumor type, and immune-cell localization before meaningful testing.	Akce et al. (60); Armstrong et al. (64); NCT04114136 (65); NCT04477343 (66)	Future trials should include liver staging and tumor biomarkers. Existing studies do not test whether MASLD/MASH stage modifies ICI response.

Evidence levels indicate how directly each study type supports a link between MASLD/MASH-related liver changes and ICI response. Direct clinical evidence refers to ICI outcome data linked to liver metastasis, not to validated MASLD/MASH-based treatment selection. Translational human evidence includes human tissue, pathology, imaging, or molecular profiling data, sometimes supported by mechanistic experiments. Animal-model evidence refers to diet-induced fatty liver or steatohepatitis models and pathway-intervention studies. Mechanistic evidence refers to related NET, stromal, or immune-cell localization pathways that have not been validated in MASLD/MASH-staged gastrointestinal liver metastasis cohorts. Potential treatment approaches refer to strategies that should be tested together with liver disease stage and tumor biomarkers. GI, gastrointestinal; HGP, histopathological growth pattern; ICI, immune checkpoint inhibitor; ICIs, immune checkpoint inhibitors; MASLD, metabolic dysfunction-associated steatotic liver disease; MASH, metabolic dysfunction-associated steatohepatitis; NET, neutrophil extracellular trap; PD-1, programmed cell death protein 1.

## 2 MASLD, MASH, and disease stage

### 2.1 MASLD versus MASH

MASLD and MASH are related but not identical terms. The 2023 multisociety Delphi consensus replaced non-alcoholic fatty liver disease (NAFLD) with MASLD and non-alcoholic steatohepatitis (NASH) with MASH, while retaining steatohepatitis as a distinct disease state (7). The 2024 European multisociety Clinical Practice Guidelines define MASLD as steatotic liver disease associated with at least one cardiometabolic risk factor (8). In this review, MASLD refers to the broader background of metabolic liver disease, whereas MASH refers to the inflammatory steatohepatitic form characterized by hepatocellular injury and inflammatory activity. This state may also be accompanied by fibrosis.

This distinction is important because not all metabolic liver disease has the same effect on the liver and tumor environment. A liver with simple fat accumulation is different from a liver with active steatohepatitis, immune-cell recruitment, and matrix remodeling. Several mechanisms discussed later in this review, including hepatocyte injury, chemokine release, myeloid-cell recruitment, and stromal remodeling, are more closely related to active steatohepatitis than to isolated steatosis (10, 11). Therefore, evidence from simple steatosis, active MASH, fibrosis, or cirrhosis should not be treated as equivalent. When older studies use the terms NAFLD or NASH, we keep the original terminology when describing those studies, but interpret their findings within the current MASLD and MASH terminology.

A further interpretive caution concerns diagnostic heterogeneity across the cited literature. Much of the evidence synthesized here predates the 2023 MASLD nomenclature and relied on heterogeneous NAFLD/NASH definitions—histological scoring (e.g., NAS or SAF), imaging-based steatosis thresholds, serum surrogate scores (e.g., fatty liver index, NAFLD fibrosis score), or chart-based diagnoses—that are not interchangeable. Studies also differed in whether they captured simple steatosis, steatohepatitis, or fibrosis, and few required the cardiometabolic criteria now central to MASLD. These differences introduce classification bias: an association reported under a broad ‘NAFLD’ label may reflect steatosis, steatohepatitis, or fibrosis to varying degrees across cohorts. We therefore interpret older findings within current MASLD/MASH terminology while noting that apparent inconsistencies between studies may partly reflect divergent definitions rather than true biological discordance.

### 2.2 Stages of metabolic liver disease

Disease stage is important when considering the possible effect of MASLD/MASH on liver metastasis. Simple steatosis, active steatohepatitis, fibrosis, and cirrhosis represent different liver conditions rather than different labels for the same process. Histological scoring systems separate steatosis, lobular inflammation, hepatocellular ballooning, and fibrosis because these features reflect related but distinct biological changes (12, 13). This distinction is especially important in liver metastasis. Lipid accumulation, inflammatory activity, sinusoidal remodeling, and matrix stiffness can affect metastatic colonization, immune-cell entry, and metastatic growth in different ways.

In this review, simple steatosis is considered mainly as a lipid-rich liver condition, without assuming the inflammatory or fibrotic features of MASH. Active MASH is considered an inflammatory and lipotoxic condition in which hepatocyte injury and myeloid-cell recruitment may support metastatic growth. Early fibrosis is considered a transitional stage in which matrix remodeling begins to alter liver architecture. Advanced fibrosis and cirrhosis are treated as advanced chronic liver disease. In advanced cirrhosis, initial tumor-cell seeding in the liver may be less likely, while lymphocyte access to established lesions may also be limited. This difference is discussed in later sections.

The evidence is therefore organized by study type—clinical outcomes, human tissue studies, animal models, and possible mechanisms. Throughout this review, therefore, MASLD/MASH stage is treated as a background liver variable that should be measured directly in future studies, not as a validated predictive biomarker for ICI treatment selection. This single qualification governs the evidence discussed in all subsequent sections and is not repeated in full thereafter. The main evidence levels used in this review are summarized in Table 1.

## 3 Hepatic immune tolerance and immunotherapy response in liver metastases

### 3.1 Hepatic immune tolerance

The liver has a unique immune environment. It is constantly exposed to portal venous antigens, microbial products, and gut-derived inflammatory signals, and has mechanisms that limit excessive immune activation (3, 14). This tolerant environment is maintained by several liver cell populations, including hepatocytes, hepatic macrophages, sinusoidal endothelial cells, and other resident immune cells (14, 15). In patients with liver metastases, these immune-regulatory mechanisms can influence how antitumor T cells are primed, recruited, and maintained (2, 3).

Metastatic tumor cells therefore enter a liver environment already primed to tolerate foreign antigens and limit immune activation (3, 14, 16). In this setting, tumor cells interact with sinusoidal antigen-presenting cells, macrophages, suppressive cytokine signals, and the hepatic vasculature. These factors can help metastatic cells survive under immune pressure. For this reason, liver metastasis should be considered not only as a marker of advanced disease, but also as a liver site with distinct immune features.

### 3.2 Liver metastasis-induced systemic T-cell deletion

Liver metastases may also weaken antitumor immunity outside the liver. Clinical and experimental data suggest that hepatic lesions can reduce the efficacy of immune checkpoint blockade by depleting tumor-specific effector T cells. In a key study, Yu et al. showed that patients with liver metastases derived less benefit from immunotherapy and that liver metastases reduced systemic antitumor immunity in preclinical models. In that study, tumor-specific CD8-positive T cells were recruited to the liver and underwent macrophage-mediated elimination. This process reduced the effector T cells that checkpoint inhibitors need to work (2).

These data change how liver involvement should be interpreted in immunotherapy studies. Liver metastasis is not only a site where resistant tumor deposits grow. It may also reduce the number of tumor-reactive T cells available for systemic antitumor immunity. If tumor-specific effector cells are deleted or markedly reduced before treatment, blocking inhibitory receptors may have limited benefit, even when the checkpoint inhibitor reaches the tumor. This is one explanation for why patients with liver metastases often have poorer outcomes after ICI therapy in several clinical analyses (2, 5, 6, 17).

The same pattern is seen in colorectal cancer. A secondary analysis of the Canadian Cancer Trials Group CO.26 trial found that liver metastases were associated with worse outcomes in patients with refractory metastatic colorectal cancer treated with durvalumab plus tremelimumab or best supportive care. Patients without liver metastases had better progression-free survival and a higher disease control rate with immune checkpoint blockade. However, this analysis was exploratory and should not be used as definitive treatment guidance (5). The data support stratification by liver involvement in future immunotherapy trials and further study of why hepatic lesions may respond less well to systemic immune activation (18).

### 3.3 Relevance to gastrointestinal cancers

Liver metastasis is particularly important in gastrointestinal cancers because the liver is a common and clinically important metastatic site, especially in colorectal cancer and PDAC (1). In colorectal cancer, immune checkpoint blockade is clearly effective in mismatch repair-deficient or microsatellite instability-high disease, but most microsatellite-stable tumors remain poorly responsive (4, 5). Liver involvement may add an organ-level barrier to ICI response in this less immunogenic tumor subtype.

PDAC has a different but related pattern of resistance. Its immune microenvironment is often characterized by dense stroma, limited T-cell infiltration, and suppression mediated by myeloid cells and fibroblasts (19). In this setting, liver metastasis may compound an already immunosuppressive tumor microenvironment rather than act as the only barrier to ICI response. Gastric and gastroesophageal cancers require separate interpretation because immunotherapy response is influenced by PD-L1 combined positive score, mismatch repair status, HER2 status, and other tumor-intrinsic factors. Epstein–Barr virus-associated tumors can be more immunogenic, but this feature should not be treated as a uniform predictor of ICI benefit (20). Biliary tract cancers also require caution. They arise within the hepatobiliary system and are treated within a distinct hepatobiliary cancer approach, so they should not be grouped uncritically with secondary liver metastases from gastrointestinal tumors (21).

### 3.4 Immune conditions required for checkpoint blockade

ICIs do not directly kill tumor cells. Their efficacy depends on tumor-reactive T cells that are present, viable, and capable of functional recovery (22). The cancer-immunity cycle captures this logic: effective antitumor immunity requires antigen release and presentation, T-cell priming, migration to tumors, tumor infiltration, recognition of malignant cells, and cytotoxic killing (23). Effective PD-1/PD-L1 or CTLA-4 blockade therefore requires adequate antigen presentation, recruitment and survival of effector CD8-positive T cells, relief from dominant myeloid or stromal suppression, and access of lymphocytes to malignant cells (2, 24–26). These requirements are particularly relevant in liver metastases, where liver immune tolerance, macrophage activity, and stromal organization may reduce the effector T cells needed for ICI efficacy. Therefore, reduced ICI response in liver metastases could reflect not only checkpoint ligand expression, but also loss of viable effector T cells, myeloid-cell suppression, or limited tumor–T-cell contact.

Liver metastasis is therefore discussed as a common clinical setting across gastrointestinal cancers, while recognizing important differences among tumor types. The immune effect of liver involvement likely differs by tumor type, baseline immunogenicity, stromal features, and treatment-selection biomarkers.

## 4 Effects of MASLD/MASH on the liver metastatic microenvironment

### 4.1 Lipid overload and tumor metabolic adaptation

A steatotic liver may do more than supply extra lipids to tumor cells. It can also provide metabolic conditions that support tumor growth. In experimental fatty liver models using breast cancer and melanoma cells, Li et al. showed that metastatic tumor cells stimulated triglyceride lipolysis in neighboring steatotic hepatocytes. The released fatty acids were then taken up by cancer cells through FATP1 and used for mitochondrial oxidation to support metastatic growth. Human liver metastasis samples in the same study showed a similar spatial relationship between steatotic hepatocytes and tumor cells. This fatty-acid transfer mechanism has not yet been tested in gastrointestinal cancers and should not be assumed to operate in colorectal or other gastrointestinal liver metastases (27).

Fatty liver can also alter EGFR-related signaling in metastatic tumor cells. Zhang et al. reported that the NAFLD metabolic microenvironment increased FASN-dependent *de novo* palmitate synthesis in metastatic colorectal cancer cells. This increased palmitate availability promoted EGFR palmitoylation, improved EGFR protein stability and plasma membrane localization, and supported colorectal cancer stemness and hepatic colonization. Its contribution to ICI resistance in MASLD-associated liver metastasis remains unknown (28).

**Figure 2:**
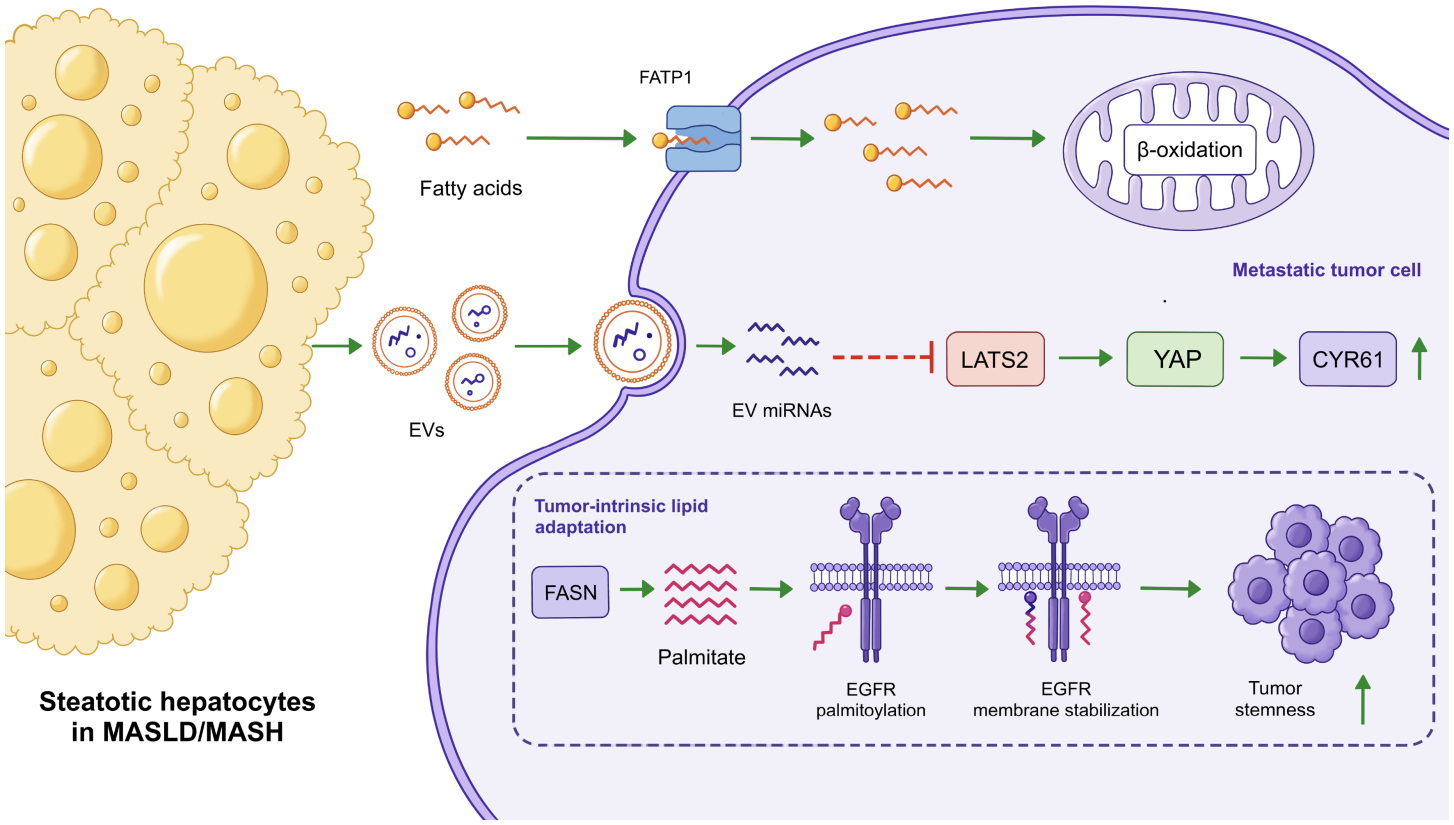
Metabolic interactions between steatotic hepatocytes and metastatic tumor cells. Steatotic hepatocytes in MASLD/MASH may fuel tumor cell growth and alter their signaling in the liver through three mechanisms. First, fatty acids released from steatotic hepatocytes can be taken up by tumor cells through FATP1 and used for mitochondrial *β*-oxidation; this pathway was shown in breast cancer and melanoma models and has not been tested in gastrointestinal cancers. Second, hepatocyte-derived EV miRNAs can suppress LATS2 in tumor cells and activate the YAP–CYR61 pathway. Third, tumor cells can increase FASN-dependent palmitate synthesis, leading to EGFR palmitoylation, EGFR membrane stabilization, and tumor stemness. The EV/YAP and FASN/EGFR pathways have been studied in colorectal liver metastasis models. Their direct relevance to ICI response remains unproven. CYR61, Cysteine-rich angiogenic inducer 61; EGFR, Epidermal growth factor receptor; EV, Extracellular vesicle; FASN, Fatty acid synthase; FATP1, Fatty acid transport protein 1; ICI, Immune checkpoint inhibitor; LATS2, Large tumor suppressor kinase 2; MASLD, Metabolic dysfunction-associated steatotic liver disease; MASH, Metabolic dysfunction-associated steatohepatitis; YAP, Yes-associated protein.

Hepatocytes exposed to lipid stress may also communicate with tumor cells through extracellular vesicles (EVs). Wang et al. showed that fatty liver increased Rab27a-dependent EV release from hepatocytes. These vesicles delivered microRNAs that suppressed LATS2 in colorectal cancer cells, thereby increasing YAP activity. YAP activation was associated with CYR61 expression and M2 macrophage infiltration in colorectal liver metastases. This pathway connects hepatocyte lipid stress and tumor signaling to an immune-suppressed environment around liver metastases. It is relevant to MASLD-associated colorectal liver metastasis, but its predictive value for ICI response has not yet been tested (29). The main hepatocyte–tumor metabolic pathways discussed in this section are summarized in Figure 2.

Importantly, the metabolic pathways summarized here were, in most cases, characterized for their effects on tumor-cell metabolism, signaling, and growth rather than for direct effects on antitumor immunity within the same experimental system. The FATP1-mediated fatty-acid transfer described by Li et al. and the FASN/EGFR-palmitoylation axis described by Zhang et al. were not evaluated for accompanying changes in T-cell or myeloid-cell function, so their connection to immune escape and ICI response remains speculative. The hepatocyte-derived extracellular-vesicle pathway described by Wang et al. is a partial exception, since it linked LATS2 suppression and YAP-CYR61 activation to increased M2-like macrophage infiltration; even there, however, an immune readout was reported without testing whether it alters checkpoint-blockade efficacy. These pathways are therefore presented as candidate metabolic enablers of metastasis whose immunological consequences require direct testing in the same models.

### 4.2 Hepatocyte stress and inflammatory signaling

Lipid overload can injure hepatocytes and change inflammatory signaling in the liver. Hepatocyte injury and lipid stress can trigger chemokine release and recruit myeloid cells (11). These signals are unlikely to be identical across all stages of MASLD/MASH. They can differ according to liver disease stage, tumor type, and experimental model. In colorectal liver metastasis, the clearest fatty liver-linked pathway identified so far is the Kupffer cell-derived CXCL5/CXCR2 axis, discussed below (30).

Hepatocyte stress should be interpreted in relation to liver disease stage. It does not mean that every patient with MASLD has the same liver metastatic microenvironment. A liver with simple steatosis is different from a liver with active MASH, early fibrosis, or cirrhosis. These stages differ in inflammatory activity, stromal structure, sinusoidal organization, and immune-cell localization. For this reason, hepatocyte-derived inflammatory signaling should therefore be considered one component of liver-microenvironment remodeling and should not be assumed to occur in every patient.

The gut-liver axis may further shape this immune environment and represents a plausible but under-explored confounder. In MASLD/MASH, increased intestinal permeability, translocation of microbial products, altered bile-acid signaling, and shifts in the gut microbiome modulate hepatic innate and adaptive immunity (31); the gut microbiome is itself an emerging determinant of ICI response across several cancers (32). Microbiome- and bile-acid-related differences could therefore confound any observed association between MASLD/MASH stage and immunotherapy outcome—for example, if microbial composition co-varies with both liver-disease stage and systemic antitumor immunity. Direct evidence linking gut-derived metabolic signaling to ICI resistance specifically in MASLD-associated gastrointestinal liver metastases remains limited, so we treat the gut-liver axis as background biology and as a variable that future MASLD-stratified immunotherapy studies should measure (e.g., stool metagenomics, bile-acid profiling), rather than as a validated resistance pathway.

### 4.3 Myeloid-cell remodeling in MASLD/MASH

MASLD- and MASH-associated inflammation can alter hepatic myeloid cells in ways that favor tumor growth and immune suppression. These changes include suppressive myeloid-cell recruitment, angiogenesis, macrophage polarization, and reduced T-cell activity. A direct preclinical link between fatty liver, myeloid recruitment, and checkpoint resistance has been reported in colorectal liver metastasis models. Yang et al. found that concomitant NAFLD induced Kupffer cells to secrete CXCL5, which recruited CXCR2-positive myeloid-derived suppressor cells (MDSCs) and promoted colorectal liver metastasis. In the same study, fatty liver was associated with poor response to anti-PD-1 treatment in mice. Pharmacologic blockade of the CXCR2-dominant MDSC axis with reparixin, a C-X-C motif chemokine receptor 1/2 (CXCR1/2) inhibitor, improved the effect of anti-PD-1 therapy in mice (30).

Inflammasome signaling can also contribute to myeloid-cell changes in fatty liver. Ohashi et al. showed that high-fat diet-induced NAFLD increased colon cancer liver metastasis and promoted the accumulation of CD206-positive M2-like tumor-associated macrophages. In NAFLD livers, deletion of NLRC4 reduced metastatic tumor growth, M2 macrophage accumulation, IL-1β expression, vascularity, and VEGF expression. IL-1 receptor blockade also reduced tumor formation and M2-type macrophage polarization under fatty liver conditions. These findings support a palmitate-sensitive NLRC4–IL-1β–VEGF axis in tumor-associated macrophages. However, this evidence remains preclinical and does not directly prove ICI resistance (33).

Together, these studies suggest that fatty liver can alter both the composition and location of immune cells in liver metastases. For immunotherapy, macrophage and MDSC abundance should be interpreted together with their location, activation state, and interaction with CD8-positive T cells (34). Checkpoint blockade requires effector T cells that can enter the lesion, survive, and maintain cytotoxic function.

### 4.4 Cancer-type and genotype specificity

The effect of MASLD and MASH on liver metastasis is unlikely to be the same across all gastrointestinal cancers. Colorectal cancer, PDAC, gastric and gastroesophageal cancer, and biliary tract cancer differ in how they spread, how immunogenic they are, how much they rely on stroma, and what metabolic programs their oncogenes drive (19–21). These differences make it inappropriate to treat all gastrointestinal liver metastases as one biological category.

In colorectal cancer, microsatellite instability and mismatch repair status remain central determinants of ICI response (4). Other tumor variables, including KRAS and BRAF alterations, may also influence metastatic behavior and immune features. In this setting, MASLD or MASH should be understood as a liver-related modifier in a heterogeneous tumor disease, not as an isolated determinant of treatment response.

PDAC has a different immune and stromal background. Its immune environment is often characterized by dense stroma, low T-cell infiltration, and strong suppression mediated by myeloid cells and fibroblasts (19). In addition, PDAC-derived EVs can prime the liver pre-metastatic microenvironment through Kupffer cell and hepatic stellate cell activation. Both tumor-derived and host-derived signals therefore appear to shape PDAC liver tropism (35). Yu et al. reported that hepatic macrophage migration inhibitory factor was increased in MASLD and promoted pancreatic cancer stemness, metastatic adhesion, and liver metastasis through CD44-mediated mechanisms (36). That study does not establish MASLD as a predictor of ICI response in pancreatic cancer.

Gastric, gastroesophageal, and biliary tract cancers also require separate interpretation. In gastric and gastroesophageal cancer, immunotherapy selection and response assessment are influenced by PD-L1 combined positive score, mismatch repair status, Epstein–Barr virus association, HER2 status, and tumor mutational burden (20). Biliary tract cancers are even more distinct because they arise within the hepatobiliary system itself. Their immune environment should therefore not be treated as equivalent to secondary liver metastases from colorectal, pancreatic, or gastric cancer (21). Future MASLD/MASH-stratified studies should account for tumor type and genotype.

The strength of MASLD/MASH-ICI evidence by tumor type is summarized in Table 2.

**Table 2:** Tumor-type differences in evidence linking MASLD/MASH-related liver changes, liver metastasis biology, and ICI response.

Cancer type	Strength of MASLD/MASH-ICI evidence	Liver-metastasis/ICI context	Representative MASLD/MASH-related evidence	Principal caveat
Colorectal cancer	Strongest (still preclinical for ICI)	ICI effective in dMMR/MSI-H; MSS poorly responsive; liver metastasis reduces ICI benefit (2, 5, 6, 17)	CXCL5/CXCR2-MDSC anti-PD-1 refractoriness (30); HAS2/HA (39); NLRC4 (33); NAFLD-metastasis associations (40, 41, 44, 50)	Mechanistic data largely murine; no MASLD-staged human ICI cohort
Pancreatic ductal adenocarcinoma	Emerging	Immunosuppressive stroma; ICI largely ineffective; liver tropism via pre-metastatic niche (19, 35)	MASLD-MIF-CD44 promotes pancreatic metastasis (36)	No ICI-outcome data by MASLD/MASH stage; PDAC immune resistance multifactorial
Gastric/gastroesophageal	Limited	Response governed by CPS, MMR status, EBV, HER2, TMB (20)	No direct MASLD/MASH-stratified liver-metastasis evidence	Tumor-intrinsic biomarkers dominate; MASLD/MASH role untested
Biliary tract	Limited/distinct	Primary hepatobiliary cancers, not secondary liver metastases (21)	Not applicable as secondary liver metastasis	Should not be grouped with secondary GI liver metastases

Evidence strength reflects how directly MASLD/MASH stage has been linked to ICI response in each tumor type; all current mechanistic links are preclinical or translational. CPS, combined positive score; dMMR, mismatch repair-deficient; EBV, Epstein–Barr virus; GI, gastrointestinal; HA, hyaluronan; HAS2, hyaluronan synthase 2; HER2, human epidermal growth factor receptor 2; ICI, immune checkpoint inhibitor; MIF, macrophage migration inhibitory factor; MMR, mismatch repair; MSI-H, microsatellite instability-high; MSS, microsatellite stable; NAFLD, non-alcoholic fatty liver disease; PD-1, programmed cell death protein 1; TMB, tumor mutational burden.

### 4.5 MASLD/MASH and ICI efficacy in liver metastases

MASLD/MASH-related liver changes can affect several requirements for ICI benefit. Steatosis or steatohepatitis can increase lipid stress, hepatocyte injury signals, and myeloid-cell recruitment, whereas fibrosis can limit immune-cell access at the tumor-liver interface. These liver-related effects differ from tumor-intrinsic mechanisms such as low tumor mutational burden or impaired antigen presentation. However, the strength of evidence remains uneven. The CXCL5/CXCR2-MDSC axis and HAS2/hyaluronan-mediated stromal remodeling provide the most direct preclinical links between fatty liver-associated liver metastases and anti-PD-1 response. Other pathways, including NET-associated hepatic seeding and lipid-induced T-cell dysfunction, remain possible mechanisms that require validation in MASLD/MASH-staged gastrointestinal liver metastasis cohorts and should be interpreted under the qualification in Section 2.2 (30, 37–39).

It should be emphasized that the CXCL5/CXCR2-MDSC and HAS2/hyaluronan axes derive predominantly from murine models and tumor-cell systems. The corresponding biomarkers have not been systematically validated in human MASLD/MASH-staged metastatic liver tissue: to our knowledge, no published series has quantified CXCR2-positive MDSC infiltration, CXCL5 expression, or hyaluronan/HAS2 burden in human gastrointestinal liver metastases stratified by background-liver MASLD/MASH stage and linked to ICI outcome. This absence of human, stage-annotated tissue validation is precisely the gap motivating the prospective tissue-based studies proposed in Section 7.

## 5 MASLD/MASH stage and liver metastasis

### 5.1 Early steatohepatitis and liver metastasis

Early metabolic liver disease may create a liver environment that supports hepatic seeding. This state may involve increased lipid availability, hepatocyte injury, endothelial activation, and myeloid-cell recruitment before dense fibrosis becomes dominant. Clinical studies in colorectal cancer suggest a possible association between fatty liver disease and hepatic metastatic recurrence, although the evidence remains retrospective, heterogeneous, and dependent on how steatosis, fibrosis, and metastatic timing are defined. Miyata et al. reported that NAFLD was associated with a higher risk of liver metastasis after radical surgery for colorectal cancer (40). Lv and Zhang also found that NAFLD was associated with synchronous colorectal liver metastasis in newly diagnosed patients, while their analysis suggested that advanced fibrosis or cirrhosis may show a different risk pattern (41).

Experimental work is consistent with the possibility that steatotic liver tissue can favor initial tumor growth in the liver. Although this murine high-fat-diet evidence predates the current MASLD terminology, it remains useful because it separates the effects of early steatosis from those of advanced cirrhosis (42).

These observations are consistent with the mechanisms discussed above. A steatotic and inflamed liver may provide metabolic substrates, inflammatory signals, and myeloid-cell recruitment that help disseminated tumor cells survive and colonize the liver during the early phase. However, most clinical studies define fatty liver by imaging, laboratory scores, or retrospective chart review rather than paired non-tumor liver histology, and results are not fully consistent (43, 44). Differences in cancer stage, treatment exposure, metastatic timing, and fibrosis assessment also limit interpretation. Early active steatohepatitis should therefore be viewed as a possible supportive condition, not a universal driver of gastrointestinal liver metastasis.

### 5.2 Advanced fibrosis and liver metastasis

Advanced fibrosis can affect liver metastasis in more than one way. It can reduce the chance that tumor cells colonize the liver, particularly when cirrhosis is present, but it may also limit immune-cell access once metastases are established. Several clinical observations suggest that hepatic steatosis, chronic liver disease, or cirrhosis is associated with a lower incidence of colorectal liver metastasis (45–47). Cai et al. reported lower incidences of colorectal liver metastases in patients with chronically diseased livers than in those with normal livers, and Gervaz et al. found that liver metastases were uncommon among patients with colorectal adenocarcinoma and cirrhosis. The data support the possibility that severe architectural remodeling, altered portal flow, sinusoidal capillarization, and increased matrix stiffness make initial hepatic spread less efficient, at least in patients with established cirrhosis (48).

This interpretation is not universal. Kondo et al. reported that hepatic fibrosis estimated by the NAFLD fibrosis score was associated with higher liver-specific recurrence after curative colorectal cancer surgery (49). The discrepancy matters: fibrosis estimated from a non-invasive score in a postoperative cohort may not reflect the same biology as established cirrhosis or severe chronic liver disease. Fibrosis stage, etiology, vascular remodeling, residual inflammatory activity, tumor biology, and competing mortality may all influence whether the liver is more or less favorable for metastasis.

Preclinical data also argue against a simple unidirectional explanation. Yamada et al. reported that NASH inhibited colon cancer liver metastasis in a western-diet mouse model, with evidence implicating IL-6, STAT3, and SAA signaling. This suggests that steatohepatitis or early fibrosis can sometimes suppress, rather than promote, metastatic growth. In lesions that do establish within remodeled liver tissue, stromal organization may still restrict lymphocyte access and reduce direct contact between immune cells and tumor cells. However, it does not show that advanced fibrosis improves antitumor immunity or predicts better response to checkpoint blockade (50).

### 5.3 Histopathological growth patterns in liver metastases

Histopathological growth patterns describe how metastatic tumor cells grow in relation to the surrounding liver parenchyma. Consensus guidelines classify liver metastases into desmoplastic, pushing, replacement, and rarer patterns according to the tumor–liver interface (51, 52).

The replacement pattern is characterized by tumor growth along pre-existing liver architecture and sinusoidal vessel co-option. In contrast, the desmoplastic pattern has a stromal rim that separates tumor cells from adjacent liver parenchyma and is often associated with angiogenesis and immune-cell accumulation at the tumor–liver interface (51, 53). Stremitzer et al. showed that desmoplastic colorectal liver metastases were associated with an inflamed immune phenotype, whereas replacement growth was associated with poorer recurrence-free survival in their cohort (54). Other studies have also linked histopathological growth patterns with immune features in colorectal liver metastases, including immunoscore-related differences (55–57).

The data support the use of growth patterns to describe the local relationship between metastatic tumors and liver tissue. However, they do not prove that MASLD- or MASH-related fibrosis directly drives metastases toward a desmoplastic pattern. A more cautious interpretation is that progressive liver remodeling could be associated with stromal-rich tumor–liver interfaces in some lesions. By contrast, replacement growth may reflect a different growth pattern based on vessel co-option and preservation of liver architecture. Whether MASLD or MASH stage predicts histopathological growth pattern in gastrointestinal liver metastases remains unresolved.

We emphasize that no study has yet directly demonstrated that MASLD or MASH stage determines histopathological growth-pattern subtype in gastrointestinal liver metastases. The proposed link between progressive liver remodeling and desmoplastic interfaces is inferential, drawn from separate literatures on growth-pattern biology and on hepatic fibrosis, and should be regarded as hypothesis-generating until tested in cohorts with paired background-liver staging and growth-pattern scoring.

### 5.4 Stage-dependent effects: synthesis and implications

The available data suggest that the effect of MASLD/MASH on liver metastasis may differ by disease stage. In early metabolic liver disease, steatosis and active inflammation may help disseminated tumor cells colonize the liver by providing lipid substrates, hepatocyte stress signals, endothelial activation, and myeloid-cell recruitment. In advanced chronic liver disease, architectural remodeling is associated with reduced initial hepatic colonization, particularly when cirrhosis, altered portal hemodynamics, and severe sinusoidal remodeling are present. Once lesions are established in a remodeled liver, stromal organization and histopathological growth pattern can influence whether cytotoxic lymphocytes can enter tumor nests and contact tumor cells.

Clinical studies linking NAFLD with synchronous or metachronous colorectal liver metastasis support a possible role for early fatty liver in helping tumor cells colonize the liver. In contrast, Yamada et al. reported that more advanced NASH-like liver changes can suppress colon cancer liver metastasis through an IL-6–STAT3–SAA-related program (40, 41, 50). These findings should not be reduced to the claim that MASLD simply promotes or prevents liver metastasis. A more cautious interpretation is that steatosis, active steatohepatitis, fibrosis, and cirrhosis can affect metastatic growth in different ways. Future cohorts should therefore measure hepatic fat burden, inflammatory activity, fibrosis stage, portal hemodynamic changes, and histopathological growth pattern, rather than grouping all patients into a single fatty-liver category (51, 54, 55).

Timing is a key limitation because MASLD progression is not synchronized with cancer dissemination. A single biopsy or imaging time point may misclassify the liver condition present during hepatic spread. This question should be tested in longitudinal cohorts with serial assessment of hepatic fat burden, inflammatory activity, and fibrosis, paired with background liver histology when available. Such studies should test whether MASLD/MASH stage at the time of metastasis is associated with hepatic seeding or immune-cell access.

**Figure 3:**
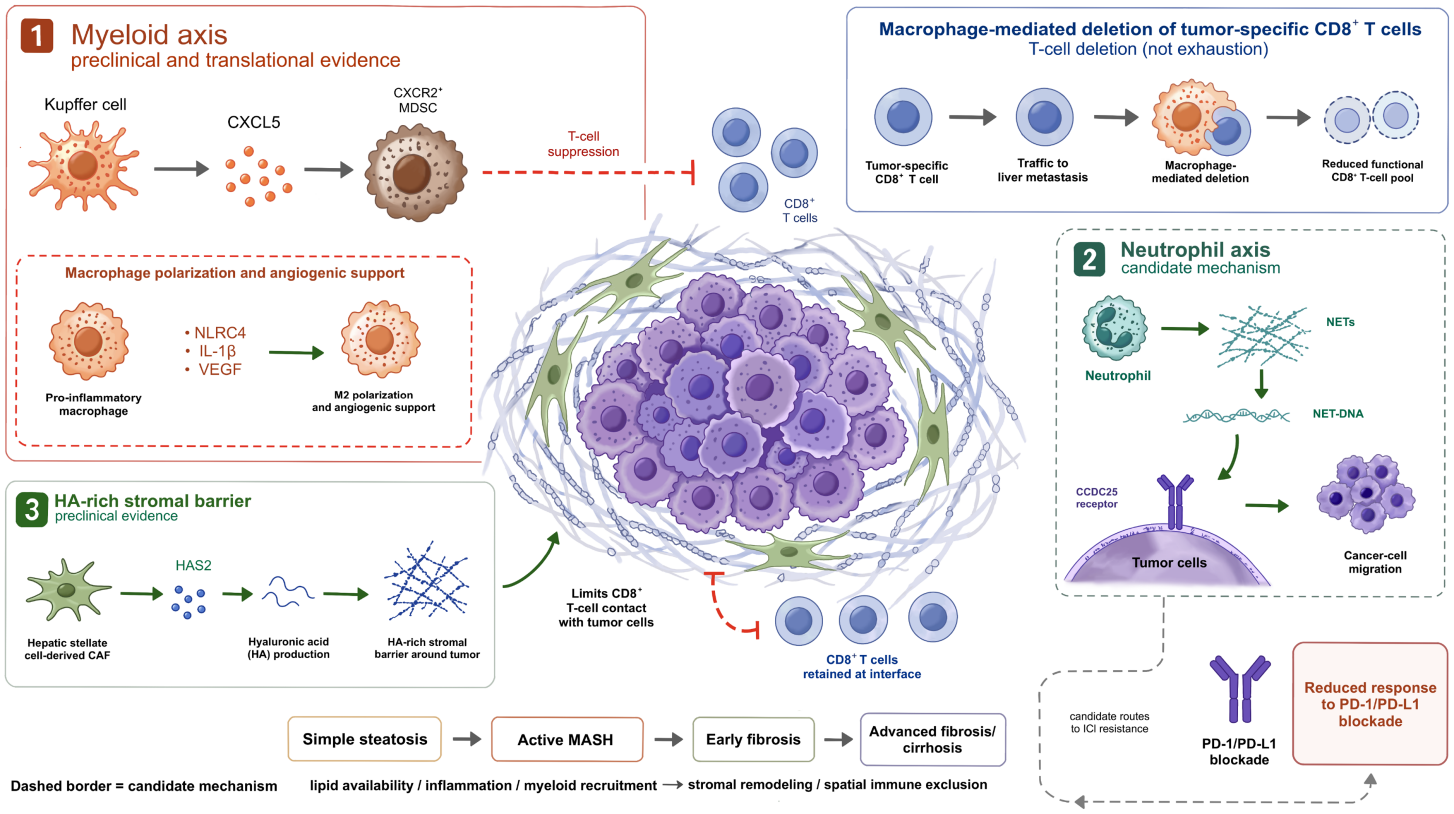
Possible links between MASLD/MASH and reduced ICI response in gastrointestinal liver metastases. The myeloid pathway shows how fatty liver drives Kupffer cells to release CXCL5, which recruits CXCR2⁺ MDSCs that suppress CD8⁺ T cells and promote macrophage polarization. Liver metastases can also reduce the tumor-specific CD8^+^ T-cell pool through macrophage-mediated T-cell elimination; this process is linked to liver metastasis but has not been shown to be MASLD/MASH-specific. NET-DNA/CCDC25 signaling is shown as a possible driver of cancer-cell migration into the liver. HAS2-driven hyaluronan deposition around the tumor is shown as a possible stromal barrier that traps CD8^+^ T cells at the interface. The lower axis represents liver disease stages, not a fixed disease sequence. Steatosis and active MASH can increase lipid availability, inflammation, and myeloid recruitment, whereas fibrosis can promote stromal remodeling and impair CD8^+^ T-cell localization. These links have not yet been tested in cohorts with MASLD/MASH staging and ICI outcome data. CAF, cancer-associated fibroblast; CCDC25, coiled-coil domain-containing protein 25; CD8, cluster of differentiation 8; CXCL5, C-X-C motif chemokine ligand 5; CXCR2, C-X-C motif chemokine receptor 2; HA, hyaluronan; HAS2, hyaluronan synthase 2; ICI, immune checkpoint inhibitor; IL-1β, interleukin-1 beta; MASLD, metabolic dysfunction-associated steatotic liver disease; MASH, metabolic dysfunction-associated steatohepatitis; MDSC, myeloid-derived suppressor cell; NET, neutrophil extracellular trap; NET-DNA, neutrophil extracellular trap-derived DNA; PD-1, programmed cell death protein 1; PD-L1, programmed death-ligand 1; VEGF, vascular endothelial growth factor.

These stage-dependent effects—on metastatic seeding, on immune-cell access to established lesions, and on the resulting requirements for ICI—are summarized in Table 3; the lower axis of Figure 3 depicts the same disease-stage spectrum.

**Table 3:** Stage-dependent effects of MASLD/MASH on liver metastasis and implications for immune checkpoint inhibition.

Liver stage	Predominant tissue features	Effect on metastatic seeding/colonization	Effect on immune-cell access to established lesions	Net implication for ICI requirements	Representative evidence	Certainty/caveat
Simple steatosis	Lipid-laden hepatocytes; minimal inflammation/fibrosis	May facilitate early seeding via lipid availability and metabolic support (FATP1; FASN/EGFR; EV/YAP)	Largely preserved	Metabolic support may aid colonization; little direct effect on T-cell access	Li (27); Zhang (28); Wang (29); NAFLD-metastasis associations (40, 41, 44)	Mechanisms mostly preclinical/non-GI; clinical associations heterogeneous
Active MASH/early steatohepatitis	Hepatocyte injury; myeloid recruitment; inflammatory signaling	May favor seeding via inflammation and myeloid recruitment (CXCL5/CXCR2-MDSC; NLRC4-IL-1β-VEGF)	Possible early impairment via myeloid suppression	Increased myeloid suppression; possible reduction in effector T-cell function	Yang (30); Ohashi (33)	Preclinical murine NAFLD models
Early fibrosis	Onset of stromal/matrix remodeling	Transitional; remodeling begins to alter architecture	Beginning of access limitation (stromal rim; hyaluronan)	Emerging stromal barrier; mixed/uncertain effects	Yang (39); HGP literature (51–57)	Inferential; not stage-validated
Advanced fibrosis/cirrhosis	Architectural distortion; altered portal flow; sinusoidal capillarization; matrix stiffness	May reduce initial seeding (especially cirrhosis)	May impair lymphocyte access to established lesions	Fewer lesions, but where present, immune exclusion may blunt ICI	Cai (45); Gervaz (46); Murono (47); contrast Kondo (49), Yamada (50)	Conflicting; established cirrhosis does not equal score-estimated fibrosis

Stages represent a continuum, not a fixed sequence. All entries are hypothesis-generating; none has been validated in gastrointestinal liver-metastasis cohorts staged for MASLD/MASH with linked ICI-outcome data. EGFR, epidermal growth factor receptor; EV, extracellular vesicle; FASN, fatty acid synthase; FATP1, fatty acid transport protein 1; HGP, histopathological growth pattern; ICI, immune checkpoint inhibitor; IL-1β, interleukin-1 beta; MASH/MASLD as above; MDSC, myeloid-derived suppressor cell; NAFLD, non-alcoholic fatty liver disease; YAP, Yes-associated protein.

## 6 Potential mechanisms linking MASLD/MASH to reduced ICI response

### 6.1 Lipid stress and T-cell deletion can reduce effector T-cell availability

Checkpoint blockade requires viable tumor-reactive effector T cells that can respond to treatment. In MASLD- or MASH-associated liver metastases, this effector T-cell population may be reduced in two related but distinct ways: lipid-associated T-cell dysfunction within the tumor microenvironment and liver metastasis-associated T-cell deletion. These processes should not be described simply as T-cell exhaustion. By contrast, ferroptotic vulnerability and macrophage-mediated deletion suggest loss of effector T-cell viability, which may leave too few cells for PD-1 or PD-L1 blockade to restore.

Lipid stress provides one possible route to reduced T-cell function. Ma et al. showed that CD36-mediated fatty-acid uptake induced lipid peroxidation and ferroptotic vulnerability in intratumoral CD8-positive T cells, reducing their effector function and antitumor activity (37). This study was not specific to gastrointestinal liver metastases or MASLD-stratified clinical cohorts. However, it supports the idea that lipid-rich tumor environments can weaken cytotoxic T cells. In steatotic liver, this mechanism is biologically plausible because metastatic lesions grow near hepatocytes and stromal cells with altered lipid handling, but it remains clinically untested.

A second route is liver metastasis-associated deletion of tumor-specific T cells. Yu et al. showed that liver metastases can recruit antigen-specific CD8-positive T cells and promote their macrophage-mediated elimination, thereby limiting immunotherapy efficacy in experimental models and clinical analyses (2). Although not MASLD-specific, this mechanism is central to this review because it shows that liver metastasis can regulate systemic antitumor immunity. In MASLD or MASH, lipid stress and liver metastasis-associated T-cell deletion may lead to the same problem: too few viable effector T cells for checkpoint blockade to restore an effective antitumor response.

**Figure 4:**
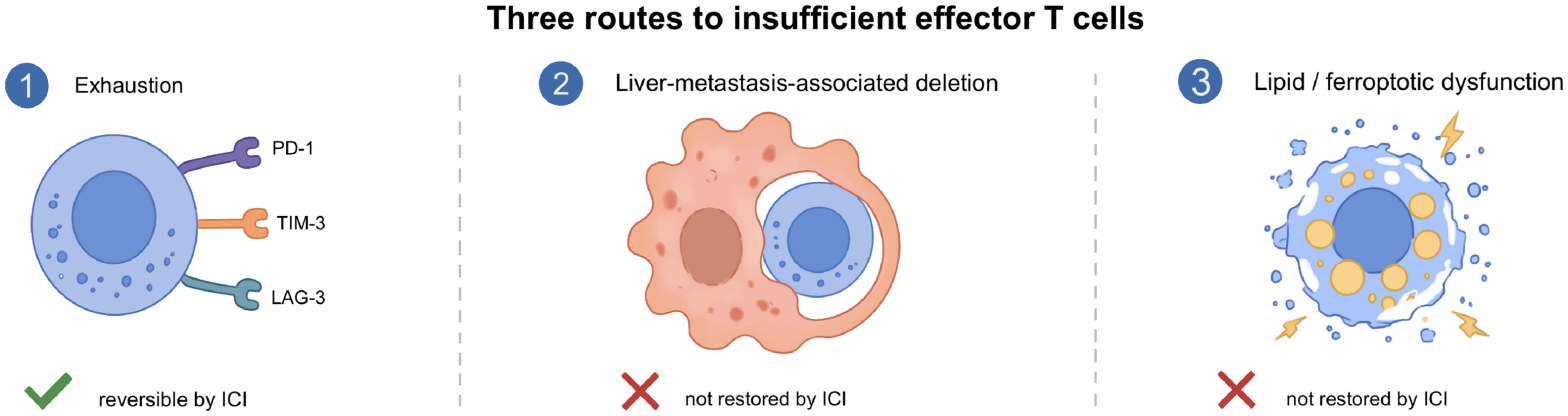
Three routes to an insufficient effector T-cell pool. A reduced pool of functional tumor-reactive CD8-positive T cells can arise through three distinct routes with different implications for checkpoint blockade: (1) exhaustion, with high expression of inhibitory receptors (PD-1, TIM-3, LAG-3), which leaves cells viable and is potentially reversible by ICI; (2) liver-metastasis-associated deletion, in which macrophages physically eliminate tumor-specific CD8-positive T cells, which is not restored by ICI; and (3) lipid/ferroptotic dysfunction, in which CD36-mediated lipid uptake drives lipid peroxidation and ferroptotic loss of viable cells, which is also not restored by ICI. Only exhausted (viable) cells can be re-engaged by checkpoint blockade. These mechanisms derive predominantly from preclinical and murine models and have not been validated in human gastrointestinal liver-metastasis cohorts staged for MASLD/MASH. CD8, cluster of differentiation 8; CD36, cluster of differentiation 36 (fatty-acid translocase); ICI, immune checkpoint inhibitor; LAG-3, lymphocyte-activation gene 3; PD-1, programmed cell death protein 1; TIM-3, T-cell immunoglobulin and mucin domain-containing protein 3.

These three routes to an insufficient effector pool—reversible exhaustion versus liver-metastasis-associated deletion and lipid-driven ferroptotic dysfunction—are contrasted in Figure 4. The distinction is clinically consequential: exhausted but viable cells may be re-engaged by checkpoint blockade, whereas deleted or ferroptotic cells leave too few targets for ICI to restore.

### 6.2 CXCL5/CXCR2-mediated myeloid suppression

The CXCL5/CXCR2 axis is one of the clearest preclinical links between fatty liver, colorectal liver metastasis, and anti-PD-1 response. Yang et al. reported that concomitant NAFLD induced F4/80-positive Kupffer cells to secrete CXCL5, which recruited CXCR2-positive MDSCs and promoted colorectal liver metastasis. In the same study, colorectal liver metastases in fatty liver were refractory to anti-PD-1 treatment. Blocking the CXCR2-dominant MDSC axis with reparixin, a C-X-C motif chemokine receptor 1/2 (CXCR1/2) inhibitor, improved the response to anti-PD-1 therapy in mice (30).

This pathway is notable because it links fatty liver to suppressive myeloid recruitment and anti-PD-1 resistance, not only to metastatic growth. MDSCs can suppress T-cell proliferation, cytokine production, and cytotoxic activity through oxidative stress, arginine metabolism, suppressive cytokines, and direct cell–cell interactions (25). In liver metastasis, these effects may be reinforced by the tolerant liver sinusoidal environment and by tumor-induced macrophage changes.

The evidence remains preclinical; human tissue validation is lacking (see Section 4.5), and the pathway has not been shown to predict ICI benefit in patients. These findings support testing in MASLD- or MASH-annotated colorectal liver metastasis cohorts, under the general treatment-selection qualification in Section 2.2.

### 6.3 Neutrophil extracellular traps and antitumor immunity

Neutrophil extracellular traps (NETs) may link inflammation and hepatic spread. Yang et al. identified CCDC25 as a receptor for NET-derived DNA (NET-DNA) and showed that NET-DNA promoted cancer-cell migration and hepatic seeding. Their study also reported abundant NETs in liver metastases from patients with breast and colon cancers (38).

They are relevant to gastrointestinal liver metastasis because colon cancer was included and because the liver was a major site of NET accumulation. However, they should be presented as evidence for metastasis biology rather than as direct proof that NETs or CCDC25 predict ICI response or resistance in MASLD/MASH-associated liver metastases. In MASLD and MASH, NETs are best discussed as a possible link between steatohepatitic inflammation and early hepatic spread. This requires validation in MASLD- or MASH-staged specimens with NET markers, stromal features, and immunotherapy outcomes.

### 6.4 Fibrotic remodeling and impaired tumor–T-cell contact

In fibrotic or desmoplastic liver metastases, reduced ICI response may result from limited immune-cell access rather than from absence of checkpoint targets alone. Effector T cells may accumulate at the tumor–liver interface but fail to enter tumor nests or make effective contact with malignant cells (58). Histopathological growth pattern studies support the idea that the tumor–liver interface contains useful biological information. Consensus guidelines define desmoplastic, pushing, and replacement growth patterns according to the architecture of this interface (51).

Immune-cell distribution may differ across these patterns. Stremitzer et al. found that the desmoplastic growth pattern in colorectal liver metastases was associated with an inflamed immune phenotype, whereas non-inflamed phenotypes were associated with shorter recurrence-free survival (54). Integrated multi-omics analysis of liver metastases further showed that immune microenvironment subtypes differ across metastatic lesions. SLC2A1-associated programs may promote immune suppression and immune-desert phenotypes through increased SPP1-positive macrophage abundance and inhibitory macrophage–T-cell interactions (59). These data also show that growth pattern and immune phenotype are not identical. The metastatic interface can influence immune-cell access, but morphology alone does not prove response or resistance to checkpoint blockade. Desmoplasia and immune exclusion are not the same thing. A stromal rim may reflect immune containment in some lesions and immune exclusion in others, depending on whether cytotoxic cells enter tumor nests or remain confined to the margin (24).

Recent studies also point to a matrix-related mechanism. Yang et al. found that steatotic liver promoted tumor growth through a HAS2-dependent fibrotic microenvironment. This involved hepatic stellate cell activation, collagen deposition, and hyaluronan accumulation. In this pattern, steatotic liver enhanced a fibrotic and immunosuppressive metastatic microenvironment, and inhibition of HA synthesis improved anti-PD-1 activity in mice (39). Taken together, these findings suggest that fibrosis should not be viewed only as a fixed physical barrier. Specific matrix components may actively regulate immune-cell localization, myeloid polarization, and treatment sensitivity.

This mechanism may represent a targetable barrier to immune-cell access rather than a fixed physical barrier. Advanced fibrosis or desmoplastic remodeling may reduce immune-cell access and impair tumor-T-cell contact, but this has not been validated as a MASLD-specific determinant of ICI resistance in gastrointestinal liver metastases (human tissue validation is lacking; see Section 4.5). Future studies should combine histopathological growth pattern scoring, multiplex immune mapping, fibrosis staging of the background liver, and treatment outcome data (24).

Together, these mechanisms help explain why PD-1 or PD-L1 expression alone may be insufficient in liver metastases. Checkpoint blockade requires viable tumor-reactive effector T cells, relief from myeloid suppression, and direct tumor-T-cell contact. Lipid stress, T-cell deletion, CXCL5/CXCR2-driven MDSC recruitment, NETs, and fibrotic remodeling can interfere with these requirements. These findings should guide future studies, but should not be read as establishing MASLD or MASH as a treatment-selection biomarker (see Section 2.2). The proposed links between MASLD/MASH stage, immune-cell changes, and reduced ICI response are summarized in Figure 3.

## 7 Clinical and translational implications

### 7.1 Assessment of background liver factors before treatment

Current MASLD guidelines recommend a stepwise, non-invasive approach for patients with cardiometabolic risk factors. This usually begins with blood-based fibrosis scores and, when appropriate, proceeds to imaging-based assessment of liver stiffness (8). For research on gastrointestinal liver metastasis, this assessment should be linked to studies of the metastatic immune environment.

For practical application, these measures should be separated into clinically available staging tools and exploratory research markers (Table 4). Clinically available tools that can be deployed now include FIB-4 and transient elastography (liver stiffness measurement) for fibrosis risk, MRI-PDFF and controlled attenuation parameter for hepatic fat burden, and non-tumor liver histology where clinically obtained. Exploratory markers—requiring validation before use in treatment selection include circulating MDSC frequency and CXCL5/CXCR2 activity, lipidomic and bile-acid profiles, PRO-C3, CD8-positive T-cell localization, macrophage and myeloid-cell distribution, NET markers, stromal-rim and hyaluronan features, histopathological growth pattern, and markers of macrophage-T-cell crosstalk.

**Table 4:** Clinically available versus exploratory measures for future MASLD/MASH-stratified ICI studies in gastrointestinal liver metastasis.

Domain	Clinically available tools	Exploratory research markers
Hepatic fat burden	MRI-PDFF; controlled attenuation parameter (CAP); CT liver attenuation	Hepatic lipidomics
Steatohepatitic activity	ALT/AST; liver histology where clinically obtained	Circulating inflammatory/myeloid-activation markers
Fibrosis stage	FIB-4; transient elastography (liver stiffness measurement); MR elastography; histology	PRO-C3 and other collagen neo-epitope markers (70)
Metastatic immune microenvironment	None routine	Multiplex/spatial immune mapping (CD8-positive localization, macrophage/MDSC distribution); HGP scoring; hyaluronan/stromal-rim features; NET markers; CXCL5/CXCR2 activity (59, 69)
Systemic/circulating	None routine	Circulating MDSC frequency; bile-acid profiling; stool metagenomics

Clinically available tools can be deployed now in observational cohorts; exploratory markers require validation before any treatment-selection use. ALT, alanine aminotransferase; AST, aspartate aminotransferase; CAP, controlled attenuation parameter; CD8, cluster of differentiation 8; FIB-4, fibrosis-4 index; HGP, histopathological growth pattern; ICI, immune checkpoint inhibitor; MDSC, myeloid-derived suppressor cell; MRI-PDFF, magnetic resonance imaging proton density fat fraction; NET, neutrophil extracellular trap; PRO-C3, N-terminal type III collagen propeptide.

Such an assessment would not replace established tumor-intrinsic biomarkers such as microsatellite instability, mismatch repair status, tumor mutational burden, or PD-L1 expression. Instead, it could help interpret these biomarkers in the setting of liver metastasis. At present, this approach is suitable for research, not routine treatment selection.

### 7.2 Potential therapeutic approaches

These approaches should be viewed as experimental rather than clinical recommendations. Among the approaches discussed, metabolic modulation has the most mature clinical evidence in MASLD, although not for improving ICI outcomes. The phase II NCT03800602 trial tested nivolumab plus metformin in treatment-refractory microsatellite-stable metastatic colorectal cancer. The regimen was tolerable, but it showed no evidence of efficacy (60). That negative result does not rule out a metabolic contribution to liver metastasis biology. It indicates that unselected metabolic modulation is unlikely to overcome checkpoint resistance in MSS metastatic colorectal cancer.

From a nutritional and metabolic standpoint, interventions that reduce hepatic fat and steatohepatitic activity are of particular interest as potential modifiers of the liver metastatic microenvironment. Lifestyle-based weight loss and dietary modification can reduce hepatic steatosis and, at sufficient magnitude, steatohepatitis and fibrosis; pharmacologic options now include resmetirom, a liver-directed thyroid-hormone-receptor-*β* agonist and the first agent approved for MASH with fibrosis (61), and incretin-based therapies (the GLP-1 receptor agonist semaglutide and the dual GIP/GLP-1 agonist tirzepatide) that improve steatohepatitis in randomized trials (62, 63). Whether reducing hepatic fat, inflammation, or fibrosis before or during ICI therapy alters the metastatic immune microenvironment or treatment response is untested, and the negative nivolumab-plus-metformin result indicates that unselected metabolic add-on therapy is insufficient (60). Nonetheless, these agents provide a concrete, clinically available lever to manipulate MASLD/MASH stage, making them rational candidates for biomarker-embedded studies that pair liver-directed metabolic therapy with ICIs in patients staged for MASLD/MASH.

The strongest MASLD-related preclinical immunotherapy signal in colorectal liver metastasis involves CXCL5/CXCR2-driven myeloid-cell recruitment. In fatty liver-associated colorectal liver metastasis models, Kupffer cell-derived CXCL5 recruited CXCR2-positive MDSCs and was associated with anti-PD-1 refractoriness. Blocking the CXCR2-dominant MDSC axis with reparixin, a CXCR1/2 inhibitor, improved anti-PD-1 response in mice (30). CXCR2-positive MDSCs, CXCL5, and MASLD/MASH stage should therefore be tested in colorectal liver metastasis trials of ICI combinations.

NET-directed strategies are less developed for testing with ICIs. NET biology suggests a possible therapeutic target because NET-DNA/CCDC25 signaling can promote metastatic colonization (38). However, these findings should be interpreted mainly as evidence for metastasis biology. They do not prove that NET inhibition improves ICI response in MASLD/MASH-staged gastrointestinal liver metastases (see Section 2.2). NET markers may first be useful in exploratory studies rather than for treatment selection.

Matrix-modulating approaches also require caution. HAS2-dependent HA deposition provides a plausible stromal mechanism linking steatotic liver, fibrotic tumor microenvironment, myeloid polarization, and anti-PD-1 activity in experimental models (39). However, this remains a preclinical and translational pathway without prospective clinical validation. It should not be treated as equivalent to clinical trial evidence or as interchangeable with the CXCL5/CXCR2-MDSC pathway.

### 7.3 Limitations of current clinical trials

Existing ICI combination trials show that metabolic or myeloid-targeted strategies are already being tested. However, most studies have not prospectively stratified patients by MASLD or MASH stage, hepatic fat burden, fibrosis, or immune-cell distribution in liver metastases. Relevant trials include NCT03800602, NCT04114136, NCT03473925, and NCT04477343, which test metabolic or myeloid-targeted strategies in treatment-refractory MSS metastatic colorectal cancer, selected advanced solid tumors including gastrointestinal cancers, or metastatic PDAC (60–66). These studies are relevant to the treatment approaches discussed here, but none directly tests whether MASLD or MASH stage modifies ICI response in gastrointestinal liver metastasis. Selected relevant trials and their limitations are summarized in Table 5.

**Table 5:** Selected trials of metabolic or myeloid-targeted approaches with immune checkpoint blockade.

Trial/source	Setting	Intervention	Status/result	Key limitation for this review
NCT03800602; Akce et al. (60)	Treatment-refractory MSS mCRC	Nivolumab plus metformin	Published phase II trial; tolerable, without clear efficacy signal	No prospective assessment of MASLD/MASH stage, liver fat, fibrosis, liver metastasis, or immune-cell localization.
NCT04114136	Advanced solid tumors receiving anti-PD-1-based therapy	Anti-PD-1 mAb plus metformin or rosiglitazone	Registered phase II trial; no peer-reviewed efficacy results identified	Tests a metabolic approach, but does not select patients by MASLD/MASH stage, liver metastasis, or liver tissue features.
NCT03473925; Armstrong et al. (64)	Selected advanced solid tumors, including MSS CRC, CRPC, and NSCLC	Pembrolizumab plus navarixin	Published randomized phase II trial; manageable safety, insufficient efficacy	Tests CXCR2 blockade without selection by liver metastasis, MASLD/MASH stage, or CXCR2-positive MDSC infiltration.
NCT04477343	Metastatic PDAC after initial chemotherapy without progression	SX-682 plus nivolumab	Registered phase I trial; safety and dose-finding study	Tests CXCR1/2 blockade without assessment of MASLD/MASH stage, liver fat, fibrosis, or tissue features of liver metastases.

This table summarizes selected trials relevant to the treatment approaches discussed in this review. It shows why current trials do not directly answer whether MASLD/MASH stage modifies ICI response in gastrointestinal liver metastases. Published outcomes are cited from peer-reviewed reports when available; registered studies without peer-reviewed efficacy results are cited by ClinicalTrials.gov identifier. Liver-related stratification refers to prospective assessment of MASLD/MASH stage, liver fat, inflammatory activity, fibrosis stage, histopathological growth pattern, or immune-cell localization. None of the listed trials includes prospective liver-related stratification of this kind. NCT05604560 (67) and NCT06149481 (68) are emerging SX-682-containing immunotherapy studies, but they are not included in the main comparison because they do not address MASLD/MASH stage or other liver-related variables. CRC, colorectal cancer; CRPC, castration-resistant prostate cancer; CXCR1/2, C-X-C motif chemokine receptor 1/2; CXCR2, C-X-C motif chemokine receptor 2; ICI, immune checkpoint inhibitor; MASLD, metabolic dysfunction-associated steatotic liver disease; MASH, metabolic dysfunction-associated steatohepatitis; mAb, monoclonal antibody; mCRC, metastatic colorectal cancer; MDSC, myeloid-derived suppressor cell; MSS, microsatellite-stable; NSCLC, non-small cell lung cancer; PD-1, programmed cell death protein 1; PDAC, pancreatic ductal adenocarcinoma.

The main limitation is the lack of prospective assessment of the liver status. A future trial should not simply add a metabolic or myeloid agent to PD-1 blockade in an unselected population. It should assess hepatic fat burden, inflammatory activity, fibrosis stage, liver metastasis histopathological growth pattern, and immune-cell localization before treatment. Additional early trials, including NCT05604560 and NCT06149481, extend SX-682-containing immunotherapy strategies into pancreatic and colorectal cancer settings, but they also do not solve this stratification problem (67, 68). These trials are best cited as emerging examples, not as evidence that MASLD/MASH-guided ICI combinations are already being tested.

### 7.4 A practical study design

A pragmatic prospective framework would (1) enroll patients with gastrointestinal cancer and liver metastases starting ICI-based therapy; (2) characterize the background liver before treatment using a tiered protocol—FIB-4 plus steatosis/fibrosis imaging (MRI-PDFF and elastography or MR elastography) in all patients, with non-tumor liver histology where clinically obtained; (3) characterize MASLD and fibrosis stage according to current criteria, diagnose MASH only when histology is available, and record cardiometabolic factors; (4) score histopathological growth patterns in resection specimens or other tissue containing the tumor–liver interface, where available, and use metastatic-lesion biopsy for multiplex/spatial immune mapping (CD8-positive T-cell localization, macrophage and MDSC distribution, hyaluronan and stromal-rim features, and NET markers); (5) profile circulating markers (MDSC frequency, CXCL5/CXCR2 activity, lipidomic and bile-acid panels, PRO-C3); and (6) link these to standardized ICI outcomes (objective response, progression-free and overall survival) with a prespecified analysis of whether MASLD/MASH stage modifies benefit. Such a design—feasible as a multicenter biomarker-embedded cohort or as a correlative arm within an ICI-combination trial—would convert the mechanistic hypotheses reviewed here into testable predictions.

## 8 Conclusion and future directions

MASLD and MASH should not be viewed simply as background metabolic diseases in patients with gastrointestinal liver metastases. They may represent different liver conditions that affect liver metastases and the requirements for ICI efficacy. Current evidence indicates that the liver is an active immune-regulatory organ rather than a passive metastatic site (2, 3, 14). Experimental models show that liver metastases can promote macrophage-mediated elimination of tumor-specific CD8-positive T cells, whereas clinical analyses associate liver metastases with reduced ICI benefit. MASLD- and MASH-associated liver changes may further alter this environment through inflammation, CXCL5/CXCR2-mediated myeloid recruitment, HAS2/hyaluronan-associated stromal remodeling, and changes in immune-cell localization (10, 30, 39, 69). However, current evidence is not sufficient to define MASLD or MASH stage as a validated biomarker for ICI treatment selection.

The key point is that MASLD/MASH stage should be assessed directly rather than inferred from broad fatty-liver labels. Different stages of MASLD/MASH may have different effects on liver metastasis, myeloid-cell recruitment, CD8-positive T-cell localization, and response to ICI-based combinations (30, 50, 59, 69). Future studies should prospectively assess hepatic fat burden, inflammatory activity, fibrosis stage, histopathological growth pattern, CXCR2-positive myeloid-cell infiltration, CD8-positive T-cell localization, and ICI efficacy endpoints. Future ICI combination trials should therefore measure both tumor-intrinsic biomarkers and liver disease stage before treatment.

## Glossary

ALT: alanine aminotransferase
AST: aspartate aminotransferase
CAP: controlled attenuation parameter
CPS: combined positive score
CCDC25: coiled-coil domain-containing protein 25
CD8: cluster of differentiation 8
CXCL5: C-X-C motif chemokine ligand 5
CXCR1: C-X-C motif chemokine receptor 1
CXCR1/2: C-X-C motif chemokine receptor 1/2
CXCR2: C-X-C motif chemokine receptor 2
CYR61: cysteine-rich angiogenic inducer 61
dMMR: mismatch repair-deficient
EBV: Epstein–Barr virus
EGFR: epidermal growth factor receptor
EV: extracellular vesicle
FASN: fatty acid synthase
FATP1: fatty acid transport protein 1
GI: gastrointestinal
GIP: glucose-dependent insulinotropic polypeptide
GLP-1: glucagon-like peptide-1
HA: hyaluronan
HAS2: hyaluronan synthase 2
HER2: human epidermal growth factor receptor 2
HGP: histopathological growth pattern
ICI: immune checkpoint inhibitor
ICIs: immune checkpoint inhibitors
IL-1β: interleukin-1 beta
IL-6: interleukin-6
LATS2: large tumor suppressor kinase 2
mAb: monoclonal antibody
MASLD: metabolic dysfunction-associated steatotic liver disease
MASH: metabolic dysfunction-associated steatohepatitis
mCRC: metastatic colorectal cancer
MDSC: myeloid-derived suppressor cell
MDSCs: myeloid-derived suppressor cells
MSS: microsatellite stable
MRI-PDFF: magnetic resonance imaging proton density fat fraction
NAFLD: non-alcoholic fatty liver disease
NASH: non-alcoholic steatohepatitis
NET: neutrophil extracellular trap
NET-DNA: neutrophil extracellular trap-derived DNA
NETs: neutrophil extracellular traps
PD-1: programmed cell death protein 1
PD-L1: programmed death-ligand 1
PDAC: pancreatic ductal adenocarcinoma
PRO-C3: N-terminal type III collagen propeptide
TMB: tumor mutational burden
VEGF: vascular endothelial growth factor
YAP: yes-associated protein
